# DNA polymerase kappa protects human cells against MMC-induced genotoxicity through error-free translesion DNA synthesis

**DOI:** 10.1186/s41021-016-0067-3

**Published:** 2017-01-07

**Authors:** Yuki Kanemaru, Tetsuya Suzuki, Akira Sassa, Kyomu Matsumoto, Noritaka Adachi, Masamitsu Honma, Satoshi Numazawa, Takehiko Nohmi

**Affiliations:** 1 Division of Genetics and Mutagenesis, National Institute of Health Sciences, 1-18-1 Kamiyoga, Setagaya-ku, Tokyo, 158-8501 Japan; 2 Division of Toxicology, Department of Pharmacology, Toxicology and Therapeutics, Showa University School of Pharmacy, 1-5-8 Hatanodai, Shinagawa-ku, Tokyo, 142-0064 Japan; 3 Toxicology Division, The Institute of Environmental Toxicology, 4321 Uchimoriya-machi, Joso-shi, Ibaraki 303-0043 Japan; 4 Graduate School of Nanobioscience, Yokohama City University, 22-2 Seto, Kanazawa-ku, Yokohama, 236-0027 Japan; 5 Present Addresses: Graduate School of Biomedical and Health Sciences, Hiroshima University, 1-2-3 Kasumi, Minami-ku, Hiroshima 734-8553 Japan; 6 Present Addresses: Biological Safety Research Center, National Institute of Health Sciences, 1-18-1 Kamiyoga, Setagaya-ku, Tokyo, 158-8501 Japan

**Keywords:** Translesion DNA synthesis, DNA polymerase κ, Nalm-6-MSH+, Genotoxicity assay, Mitomycin C

## Abstract

**Background:**

Interactions between genes and environment are critical factors for causing cancer in humans. The genotoxicity of environmental chemicals can be enhanced via the modulation of susceptible genes in host human cells. DNA polymerase kappa (Pol κ) is a specialized DNA polymerase that plays an important role in DNA damage tolerance through translesion DNA synthesis. To better understand the protective roles of Pol κ, we previously engineered two human cell lines either deficient in expression of Pol κ (KO) or expressing catalytically dead Pol κ (CD) in Nalm-6-MSH+ cells and examined cytotoxic sensitivity against various genotoxins. In this study, we set up several genotoxicity assays with cell lines possessing altered Pol κ activities and investigated the protective roles of Pol κ in terms of genotoxicity induced by mitomycin C (MMC), a therapeutic agent that induces bulky DNA adducts and crosslinks in DNA.

**Results:**

We introduced a frameshift mutation in one allele of the thymidine kinase (TK) gene of the KO, CD, and wild-type Pol κ cells (WT), thereby establishing cell lines for the *TK* gene mutation assay, namely TK+/- cells. In addition, we formulated experimental conditions to conduct chromosome aberration (CA) and sister chromatid exchange (SCE) assays with cells. By using the WT TK+/- and KO TK+/- cells, we assayed genotoxicity of MMC. In the *TK* gene mutation assay, the cytotoxic and mutagenic sensitivities of KO TK+/- cells were higher than those of WT TK+/- cells. MMC induced loss of heterozygosity (LOH), base pair substitutions at CpG sites and tandem mutations at GpG sites in both cell lines. However, the frequencies of LOH and base substitutions at CpG sites were significantly higher in KO TK+/- cells than in WT TK+/- cells. MMC also induced CA and SCE in both cell lines. The KO TK+/- cells displayed higher sensitivity than that displayed by WT TK+/- cells in the SCE assay.

**Conclusions:**

These results suggest that Pol κ is a modulating factor for the genotoxicity of MMC and also that the established cell lines are useful for evaluating the genotoxicity of chemicals from multiple endpoints in different genetic backgrounds of Pol κ.

**Electronic supplementary material:**

The online version of this article (doi:10.1186/s41021-016-0067-3) contains supplementary material, which is available to authorized users.

## Background

Translesion DNA synthesis (TLS) is one of the strategies to circumvent persisting DNA lesions that can lead to fork collapse and cell death. TLS is performed by specialized DNA polymerases (Pols). These Pols take over primer DNA from replicative Pols, e.g., Pol δ and Pol ε, at or before lesions and insert dNMPs opposite lesions in an error-free or error-prone manner [1–3]. After successful lesion bypass, the replicative Pols return to the primer DNA and continue whole chromosome replication [4]. The bypass process does not accomplish repair or excision of the DNA lesions. In addition, the fidelity of TLS Pols in lesion bypass depends on the polymerases used and the type of lesions encountered. Therefore, TLS is regarded as a mode of DNA damage tolerance that may cause mutations in compensation for cell survival [5].

To date, more than ten such specialized Pols involved in TLS have been identified. Among them, the Y-family Pols η, ι, κ and REV1, and the B-family Pol ζ have been intensively characterized [6, 7]. Among the Y-family Pols, Pol κ is primarily characterized by its ability to perform error-free TLS across bulky DNA adducts at *N* ^2^ position of guanine induced by benzo[*a*]pyrene-7,8-dihydrodiol-9,10-epoxide (BPDE) [8–11]. Several in vitro studies, mostly biochemical, have also demonstrated that Pol κ bypasses a wide variety of DNA lesions that are structurally unrelated, such as 7,8-dihydro-8-oxo-guanine [12–15], thymine glycol [16, 17], *N* ^3^-methyl-adenine [18] and DNA inter-strand crosslinks [19–21]. In vivo, Pol κ-deficient mice exhibited a spontaneous mutator phenotype [22]. Besides, upregulation of Pol κ was observed in tumor tissues from patients with non-small cell lung cancer [23], and overexpression of human Pol κ also confers genomic instability at the cellular level [24]. These findings implicate that Pol κ plays a role in maintaining human genome integrity.

To better understand the protective roles of Pol κ at the cellular level, we previously generated human Pol κ knockout (KO) [25] and catalytically dead (CD) mutants in the human Nalm-6-MSH+ cell line and examined cytotoxic sensitivity to various genotoxins [26]. We conducted the study with not only KO cells but also CD cells because Pol κ interacts with other proteins, such as REV1 and proliferating cell nuclear antigen PCNA. REV1 acts as a docking site for other TLS Pols and facilitating polymerase exchange [27], and PCNA interacts with various proteins involved in DNA replication and repair pathways [28]. Therefore, a simple KO of Pol κ might modulate functions of other proteins, thereby obscuring the possible catalytic protective role against genotoxic stresses. Results suggested that Pol κ protects cells against BPDE, mitomycin C (MMC), and bleomycin via its DNA polymerase activity. In addition, we shed light on a novel non-catalytic protective role of this protein against oxidative stress induced by hydrogen peroxide. These findings have emphasized the significance of Pol κ in the protection of human cells against various genotoxic stresses. However, whether the protective roles of Pol κ are attributed to error-free or error-prone TLS have yet to be clarified. Therefore, in this study, we established cell lines for the thymidine kinase (*TK*) gene mutation assay by introducing a frameshift mutation in one allele of the *TK1* in cells with altered Pol κ activity, resulting in TK+/- cells. In addition, to gain insight into chromosomal events, we formulated experimental conditions for chromosome aberration (CA) and sister chromatid exchange (SCE) assays.

To evaluate the utility of the cell lines to investigate protective roles of Pol κ in terms of genotoxicity, we exposed Pol κ wild-type (WT) TK +/- and KO TK+/- cells to MMC and investigated gene mutations and chromosomal damage. MMC is a chemotherapeutic agent that induces monofunctional adducts, and intra- and inter-strand DNA crosslinks at the *N* ^2^ position of guanine [29, 30]. Although the amounts of DNA inter-strand crosslinks are lower than those of intra-strand crosslinks or monofunctional adducts, these inter-strand crosslinks are largely responsible for the cytotoxicity of MMC [31]. Earlier studies reported that Pol κ is involved in error-free TLS across *N* ^2^-guanine inter-strand crosslinks and *N* ^2^-guanine monofunctional adducts [19, 32, 33]. However, the roles Pol κ plays to protect cells from mutagenic and clastogenic effects of multiple DNA damages induced by MMC in human cells are unclear. Therefore, we analyzed the genotoxicity of MMC in human cells with different Pol κ expression profiles by using a series of genotoxicity assays.

## Methods

### Cell lines and culture

The human pre-B cell line Nalm-6-MSH+ (WT) and its Pol κ derivatives, KO and CD cells, were established in our laboratory as described previously [25, 26, 34]. The KO cells were generated by deleting exon 6 of the *POLK*, resulting in a frameshift. The CD cells were also generated by introducing mutations directing amino acid substitutions in exon 6 of the *POLK*, D198A and E199A, which are critical to the catalytic activity of Pol κ. The cells were cultured in RPMI1640 (Nacalai Tesque, Kyoto, Japan) medium supplemented with 10% calf serum (Thermo Fisher Scientific, Waltham, MA, USA), 50 μg/mL kanamycin and 50 μM 2-mercaptoethanol in a 5% CO_2_ incubator at 37 °C.

### Chemicals

BPDE was purchased from MRI Global (Kansas City, MO, USA) and used to evaluate the *TK* gene mutation assay in the TK+/- cells established in this study because it is known that Pol κ can bypass BPDE-DNA adducts in an error-free manner [11]. MMC was purchased from Nacalai Tesque (Kyoto, Japan) and used as a test chemical because the chemotherapeutic agent induces not only bulky DNA adducts but also inter- and intra-DNA crosslinks [29, 30] which are not well-known the contribution of Pol κ in their repair processes. Dimethyl sulfoxide (DMSO) was obtained from Wako (Osaka, Japan) and used as a solvent control.

### Establishment of *TK*^+/−^ mutant cells

The gene-targeting vector for introducing a +1-bp frameshift mutation in exon 4 of the *TK1* was constructed using MultiSite Gateway Three-Fragment Vector Construction Kit (Thermo Fisher Scientific, Waltham, MA, USA) as previously described [35]. The targeting vector was linearized by *Pme*I and transfected into 2 × 10^6^ WT, KO and CD cells using Nucleofector™ I with Kit T solution (LONZA, Basel, Switzerland) according to the manufacturer’s instructions. After 48 h of incubation, the cells were transferred into a selection medium containing 0.5 μg/mL puromycin (Wako, Osaka, Japan) at a density of 2,000 cells per well in 96-well plates and cultured for two-three weeks. The resulting drug-resistant clones were subjected to PCR analysis with primers TK ex4 TG Fw and 5′-loxP TG to screen the targeted integrant at exon 4 of the *TK1*. Subsequently, the targeted +1-bp frameshift mutation induction was verified by DNA sequencing using primers TK ex4 Fw and TK ex4 Rv. For the successfully targeted integrants, the drug resistance gene was excised by transient expression of Cre recombinase via electroporation of the Cre expression vector. Excision of the drug resistance gene was confirmed by PCR with primers TK ex4 TG Fw and PGK pro. All PCR reactions were performed using KOD FX (TOYOBO, Osaka, Japan). The primer sequences used for the PCR and DNA sequencing are listed in (Additional file 1: Table S1).

### *TK* gene mutation assay

Established cell lines harboring the *TK* ^+/-^ mutation were cleansed with CHAT (C: 20 μM 2′-deoxycytidine, H: 200 μM hypoxanthine, A: 0.1 μM aminopterin, T: 17.5 μM thymidine (Sigma-Aldrich, St. Louis, MO, USA)) solution prior to use as described previously [36]. Five milliliters of cell suspensions (5 × 10^5^ cells/mL) were treated with 6.25, 12.5 and 25 nM of BPDE or 50, 100 and 200 ng/mL of MMC for 3 h at 37 °C. After treatment, the cells were centrifuged, washed once, and then re-suspended in a fresh medium at a density of 2 × 10^5^ cells/mL. We also seeded the cells into 96-well plates (2.5 cells/well) concurrently for determining plating efficiency (PE0). After a 96-h expression period, the cell suspension was seeded in 96-well plates at 2.5 × 10^4^ cells/well in the presence of 3 μg/mL trifluorothymidine (TFT) to isolate *TK* ^−/−^ mutants. We also seeded the cells into 96-well plates (2.5 cells/well) in the absence of TFT for plating efficiency determination (PE4). All 96-well plates were incubated for more than 20 days at 37 °C, and then the number of wells with colonies in PE0 and PE4 plates and *TK* ^−/−^ mutant colonies in TFT plates were scored. Mutant frequencies and relative cell survivals were calculated using previously described method [37]. Comparisons of mutant frequencies or relative cell survivals between cell lines were quantified using Student’s *t*-test. The level of statistical significance was set at *P* < 0.05.

### Mutation spectrum analysis of *TK*^−/−^ mutants

Cleansed cells treated with 200 ng/mL MMC or DMSO as described in 2.4 were washed once and immediately seeded in 96-well plates (1 × 10^5^ cells/well for cells treated with MMC and 1.25 × 10^5^ cells/well for solvent control, respectively) to obtain independent *TK* ^−/−^ mutant clones. After a 96 h culture for expression period, TFT was added to each well of the plates at a final concentration of 3 μg/mL, and the cells were incubated for more than 20 days at 37 °C. The resulting TFT-resistant clones were subjected to genomic PCR to amplify exon 4 of the *TK1* using primers TK ex4 Fw and TK ex4 Rv2 to classify the mutants as LOH type, *TK* ^−/−^ mutants harboring large deletion or inter-allelic homologous recombination (one band), or non-LOH, i.e., *TK* ^−/−^ mutants harboring intragenic mutations (two bands). The PCR products of the non-functional (gene-targeted) *TK1* allele were approximately 100 bp longer than those of the functional allele due to residual sequence of the targeting vector around the *loxP* site. For non-LOH mutants, total RNA was extracted using ISOGEN II (Nippon Gene, Tokyo, Japan) and then amplified with PrimeScript^Ⓡ^ OneStep RT-PCR Kit ver. 2 (TaKaRa, Shiga, Japan) using the primers TK c176 Fw and TK c983 Rv. Resulting cDNA sequences were analyzed with 3130 Avant Genetic Analyzer (Thermo Fisher Scientific, Waltham, MA, USA) using primers TK cDNA seq Fw1 and TK cDNA seq Fw2. In the case of RNA splicing mutants, to analyze mutations around the splicing donor or acceptor sequences, genomic DNA was also extracted using Gentra Puregene Cell Kit (QIAGEN, Hilden, Germany), and the *TK* locus was amplified by PCR using sets TK95 Fw and TK4795 Rv for exons 1–4 and TK11175 Fw and TK12940 Rv for exon 5 to 7. The amplified genomic DNA sequences were analyzed using primers TK c176 Fw for exons 1–2, gTK ex3 seq Fw for exon 3,TK ex4 Fw for exon 4, gTK ex5&6 seq Fw for exon 5, and TK c983 Rv for exon 6–7.

### Chromosome aberration assay

Five milliliter of cell suspension (2.5 × 10^5^ cells/mL) was treated with 50 and 100 ng/mL MMC for 3 h at 37 °C. The highest concentration was set to achieve reduction in RICC to 45 ± 5% of the concurrent solvent control for both cell lines in accordance with the OECD Guideline for the Testing of Chemicals TG 473. The cells were washed once and cultured in fresh medium for 24 h. Two hours prior to harvesting cells, colcemid (SERVA Electrophoresis, Heidelberg, Germany) was added to the medium at a final concentration of 0.2 μg/mL. For chromosomal preparation, cells were swollen with 0.075 M potassium chloride for 10 min at room temperature, fixed with a solution of methanol : acetic acid = 3 : 1, and then air-dried on glass slides. All slides were coded and stained with 2% (v/v) Giemsa solution (Merck, Darmstadt, Germany) for 15 min at room temperature. Two hundreds of well-spread metaphase cells per dose were scored for chromosomal aberrations including breaks, exchanges and gaps at × 1000 magnification. The differences in the number of cells with chromosome aberrations between WT and KO cells were analyzed statistically using a chi-square test. The level of statistical significance was set at *P* < 0.05.

### Sister chromatid exchange assay

Cells treated with MMC for 3 h as described in 2.6 were washed once and cultured in a fresh medium containing 2 μg/mL of 5-bromo-2′-deoxyuridine (Sigma-Aldrich, St. Louis, MO, USA) for 48 h. Colcemid treatment and chromosomal preparation were performed as described in 2.5. The slides were coded and stained with 3% (v/v) Giemsa solution containing 2% (w/v) EDTA 4Na for 5 min at 40 °C [38]. Fifty well-spread metaphase cells containing 42–46 chromosomes were examined for SCE. The average number of SCEs per cell was calculated and statistically compared between WT TK+/- and KO TK+/- cells at the same dose concentrations using Student’s *t*-test. The level of statistical significance was set at *P* < 0.05.

## Results

### Establishment of *TK* gene mutation assay in Nalm-6-MSH+ cells and Their Pol κ derivatives

**Fig. 1:**
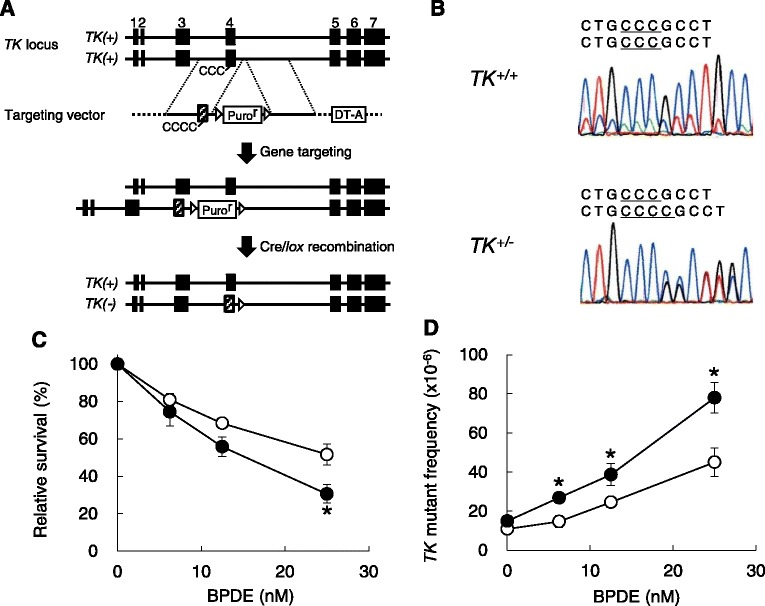
Establishment of the *TK* gene mutation assay in Nalm-6-MSH+ cells and their Pol κ derivatives. **a** Targeting strategy of knocking-in a +1-bp frameshift mutation in one allele of the *TK1*. The *TK* locus, targeting vector, targeted locus and Cre-mediated locus are shown. The black and hatched boxes represent innate exons and mutated exon 4 harboring a +1-bp frameshift, CCC to CCCC, respectively. Puro^r^ and triangles denotes the puromycin resistance gene and *loxP* sequences, respectively. DT-A is the gene encoding diphtheria toxin A used to prevent random integration of the targeting vector. **b** Genomic DNA sequence of the targeted region at exon 4 of the *TK1* in wild-type (*TK* ^*+/+*^) cells and the targeted (*TK* ^*+/−*^) clones. **c** Relative survival and (**d**) *TK* mutant frequency in WT TK+/- and KO TK+/- cells treated with BPDE. *Open* and *closed circles* indicate the results for WT TK+/- and KO TK+/- cells, respectively. Each data point is the mean value of four independent experiments and error bars indicate standard errors. * denotes statistical significance compared to WT TK+/- cells treated at the same concentrations (*P* < 0.05)

To establish the *TK* gene mutation assay, a +1-bp frameshift mutation was introduced at exon 4 of one allele of the *TK1* in WT, KO and CD cells (Fig. 1a). The targeted heterozygous frameshift mutation, CCC to CCCC, was verified by DNA sequencing (Fig. 1b). The resulting cell lines were named as WT TK+/-, KO TK+/-, and CD TK+/- based on the different expression profile of Pol κ. Those three cell lines had comparable doubling times (WT TK+/-: 21.5 ± 0.6 h, KO TK+/-: 20.9 ± 0.6 h, and CD TK+/-: 22.2 ± 0.8 h), which were not significantly different from the original WT, KO, and CD cells, respectively [26]. The spontaneous *TK* mutant frequency of KO TK+/- cells was higher than those of WT TK+/- and CD TK+/- cells, but this difference was not statistically significant (Table 1). In addition to the gene mutation assay, we also determined the baseline frequencies of CA and SCE in established cell lines. The incidences of spontaneous CA in KO TK+/- and CD TK+/- cells were comparable to those in WT TK+/- cells, whereas KO TK+/- cells exhibited a significantly higher incidence of spontaneous SCE than WT TK+/- cells (Table 1).

**Table 1:** Baseline frequencies of the *TK* ^−/−^ mutation, CA and SCE in Nalm-6-MSH+ TK+/- cells and their Pol κ derivatives

	*TK* mutant frequency^a^	CA^b^	SCE^c^
	(×10^−6^)	(%)	(incidence/cell)
WT TK+/-	7.8 ± 2.4	1.5	4.5 ± 2.2
KO TK+/-	14.1 ± 2.9	2.0	6.9 ± 2.9*
CD TK+/-	9.9 ± 3.2	1.0	4.9 ± 1.8

^a^Values are the mean ± S.E. (*n* = 3)

^b^Two hundred metaphase cells were analyzed for each cell type (*n* = 1)

^c^Fifty metaphase cells were analyzed. Data are expressed as the mean ± S.D. (*n* = 1). * denotes a significant difference compared to WT TK+/- cells

Additionally, we determined the *TK* mutant frequencies of WT TK+/- and KO TK+/- cells treated with BPDE. As a result of cytotoxicity, KO TK+/- cells exhibited hypersensitivity to BPDE compared to WT TK+/- cells (Fig. 1c). Both cell lines exhibited concentration-related increases in the *TK* mutant frequency, and KO TK+/- cells displayed a significantly higher mutant frequency than WT TK+/- cells (Fig. 1d). The enhanced mutagenicity of BPDE in Pol κ-deficient cells is consistent with previous studies using the *Hprt* gene mutation assay in mouse ES cells [8] and the *supF* forward mutation assay in the parental Nalm-6 human Pre-B cell line [25]. Therefore, we judged the *TK* gene mutation assay in the established cell lines to be functional for analyzing the genotoxicity of test articles.

### Cytotoxic and genotoxic responses to MMC

**Fig. 2:**
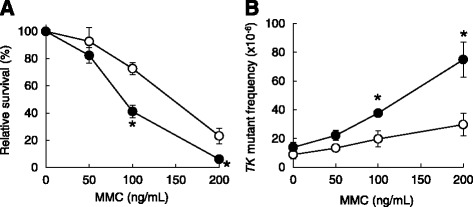
Cytotoxicity and *TK* mutant frequency in WT TK+/- and KO TK+/- cells treated with MMC. **a** Relative survival and (**b**) *TK* mutant frequency. Open and closed circles indicate the results in WT TK+/- and KO TK+/- cells, respectively. Each data point is the mean value of four independent experiments, and error bars indicate standard error. * denotes the statistical significance compared to WT TK+/- cells treated the same concentration (*P* < 0.05)

We treated KO TK+/- and WT TK+/- cells with MMC and examined cytotoxicity and genotoxicity via the *TK* gene mutation assay. In cell survival analysis, KO TK+/- cells exhibited hypersensitivity to the cytotoxic effect of MMC compared to WT TK+/- cells (Fig. 2a). The *TK* mutant frequencies of KO TK+/- cells were significantly higher than those of WT TK+/- cells at the concentrations of 100 and 200 ng/mL (Fig. 2b). These results suggest that the expression of wild-type Pol κ facilitates cell survival against MMC via error-free TLS.

### Mutation spectrum analysis

To address the sequence specificity of MMC-induced DNA lesions bypassed by Pol κ, we analyzed the mutation spectra of the spontaneous and MMC-induced *TK* ^−/−^ mutants (Table 2). A summary of all mutations is shown in (Additional file 1: Table S2). The frequencies of G:C to C:G transversions and tandem base substitutions at GpG sites were substantially increased by MMC treatment in both cell lines. The proportion of LOH mutants among KO TK+/- cells was also increased by MMC treatment.

**Table 2:** Summary of the mutation spectra of *TK* ^−/−^ mutantations^a^

	Solvent control		MMC	
	WT TK+/-	KO TK+/-	WT TK+/-	KO TK +/-
Base substitution				
Transition				
A:T → G:C	10 (36)	11 (26)	6 (6)	5 (4)
G:C → A:T	1 (4)	2 (5)	5 (5)	4 (3)
Transversion				
A:T → T:A	0 (0)	2 (5)	1 (1)	0 (0)
A:T → C:G	0 (0)	2 (5)	4 (4)	1 (1)
G:C → T:A	3 (11)	10 (23)	10 (11)	17 (13)
G:C → C:G	0 (0)	1 (2)	8 (9)	9 (7)
Tandem				
GG:CC → CT:GA	0 (0)	0 (0)	3 (3)	2 (2)
GG:CC → TT:AA	0 (0)	0 (0)	8 (9)	6 (5)
GG:CC → AT:TA	0 (0)	0 (0)	2 (2)	0 (0)
CA:GT → TG:AC	1 (4)	0 (0)	0 (0)	0 (0)
AG:TC → CT:GA	0 (0)	0 (0)	0 (0)	1 (1)
Frameshift (+1)	0 (0)	0 (0)	1 (1)	1 (1)
Frameshift (+2)	1 (4)	0 (0)	0 (0)	0 (0)
Frameshift (−1)	0 (0)	0 (0)	0 (0)	2 (2)
LOH	12 (43)	15 (35)	43 (46)	78 (62)
Unidentified	0 (0)	0 (0)	2 (2)	0 (0)
Total	28 (100)	43 (100)	93 (100)	126 (100)

^a^Data are presented as number of clones (ratio), with the results of two independent experiments are aggregated

**Fig. 3:**
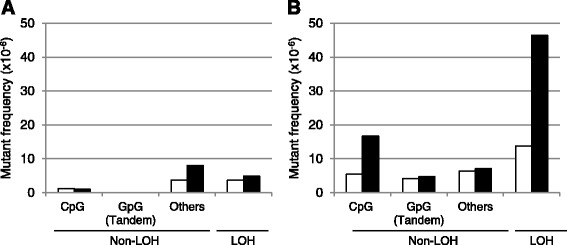
Types of mutations that contributed to *TK* mutant frequencies induced by MMC treatment. **a** Solvent control and (**b**) MMC. The *TK* mutant frequencies for each type of mutation were calculated by multiplying the mean of mutant frequency at 0 ng/mL (solvent control) and 200 ng/mL MMC taken from Fig. 2b by the ratio of each type of mutation described in Table 2. For non-LOH mutants, single base pair substitutions and frameshifts at CpG sites, tandem base pair substitutions at GpG sites, and all the other point mutations are shown separately. *White* and *black boxes* indicate the results for WT TK+/- and KO TK+/- cells, respectively

To compare the mutation spectra of MMC-induced DNA lesions in WT TK+/- and KO TK+/- cells, the absolute contribution of mutations at CpG or GpG sites and LOH was calculated by multiplying the *TK* mutant frequency at an MMC concentration of 200 ng/mL by the proportion of each type of mutations listed in Table 2 (Fig. 3). In the solvent control group, most of the *TK* ^−/−^ mutants displayed LOH and point mutations at bases other than CpG or GpG, and the ratio between LOH and point mutations was comparable between cell lines (Fig. 3a). In the MMC treatment group, however, the frequency of MMC-induced base substitution at CpG sites was increased 3.1 times in KO TK+/- cells compared to WT TK+/- cells (WT TK+/-: 5.4 × 10^−6^ versus KO TK+/-: 16.7 × 10^−6^) (Fig. 3b). In addition, the frequency of LOH mutation in KO TK+/- cells treated with MMC was 3.4 times higher than that in WT TK+/- cells (WT TK+/-: 13.8 × 10^−6^ versus KO TK+/-: 46.4 × 10^−6^). In contrast, the frequencies of the MMC-induced tandem base pair substitutions at GpG sites were comparable between the cell lines (WT TK+/-: 4.2 × 10^−6^ versus KO TK+/-: 4.8 × 10^−6^). Other base pair substitutions and frameshift mutation frequencies were not substantially increased by MMC treatment.

### Chromosome analysis

**Fig. 4:**
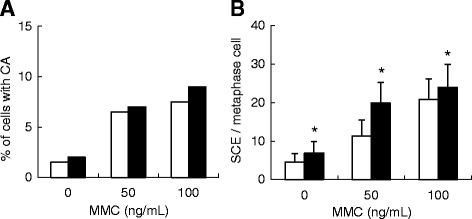
CA and SCE incidences in WT TK+/- and KO TK+/- cells treated with MMC. **a** CA assay and (**b**) SCE assay. *White* and *black boxes* represent WT TK+/- and KO TK+/- cells, respectively. Both results were drawn from single experiments. Error bars indicate the standard deviation from SCEs of 50 metaphase cells, and * denotes the statistical significance between WT TK+/- and KO TK+/- cells at the same dose concentrations (*P* < 0.05)

We measured the structural abnormalities of chromosomes for further investigation of LOH events after MMC treatment. Figure 4a and b show the results of CA and SCE assays, respectively. The incidences of both CA and SCE were elevated by MMC treatment in a concentration-dependent manner, and the frequencies of CA were comparable between WT TK+/- and KO TK+/- cells. Conversely, the incidence of SCE in KO TK+/- cells was significantly higher than that in WT TK+/- cells. SCE is known as a cytological manifestation of homologous recombination. Thus, results suggest that the increase in LOH events in KO TK+/- cells might be attributed to the induction of DNA strand breaks and the subsequent homologous recombination repair pathway.

## Discussion

In this study, to provide insights into the protective roles of Pol κ against genotoxic stresses, we have established WT TK+/-, KO TK+/- and CD TK+/- cells for conducting the *TK* gene mutation assay. The *TK* gene mutation assay is widely used in in vitro genotoxicity studies because it can detect both gene mutations and chromosomal events such as large deletions, chromosome rearrangements and mitotic recombination [39]. In terms of point mutations, mutation spectrum analysis of *TK*-deficient mutants allows us to obtain detailed information of the sequence specificity of these events. In addition, to gain insight into chromosomal damage, we have also formulated experimental conditions for the CA assay for structural damage and the SCE assay for mitotic recombination in response to DNA damage.

As previously described, the Nalm-6-MSH+ cell line which is the background cell line of KO TK+/-, CD TK+/- , and WT TK+/- cells in this study, was engineered by restoring MSH2 expression in human Nalm-6 cells which possess an exceptionally high gene-targeting efficiency, resulting in proficient mismatch repair function as well as low spontaneous mutant frequency [34]. The *TK* gene mutation assay in the WT TK+/- cells also indicated a low spontaneous *TK* mutant frequency (7.8 × 10^−6^), and this value is comparable to that seen in human lymphoblastoid TK6 cells [36]. In addition, the original Nalm-6 cell line, a parental cell line of Nalm-6-MSH+, has a normal p53 status and near diploid karyotype [40]. These properties, combined with high gene-targeting efficiency, emphasize the usefulness of this cell line as a system to investigate the functions of specific protein(s) in the protection of human genome integrity against genotoxic stresses.

**Fig. 5:**
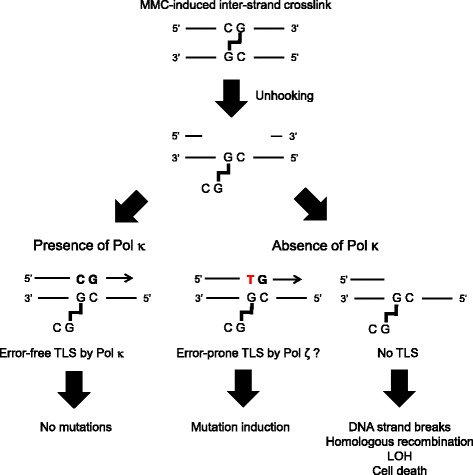
A schematic of the role of Pol κ in MMC-induced inter-strand crosslinks repair. When wild-type Pol κ is functional, error-free TLS suppresses mutation inductions (*left*). When Pol κ is deficient, other TLS Pols, such as Pol ζ, bypass the lesions in an error-prone manner (*middle*), or replication stalling leads to chromosomal alteration or cell death (*right*)

Using the WT TK+/- and KO TK+/- cells, we addressed the protective role of Pol κ against MMC genotoxicity. We did not utilize CD TK+/- cells, as KO and CD cells exhibited similar sensitivity to MMC treatment in our previous study [26]. Results from the *TK* gene mutation assay revealed a higher mutant frequency after MMC treatment in KO TK+/- cells than in WT TK+/- cells (Fig. 2b). Interestingly, there was a close relationship between the dose-related increase in the mutant frequency and cytotoxicity in both cell lines (Fig. 2). The relationship was also observed in BPDE treatment (Fig. 1c and d). The results of the mutant frequency and the survival suggest that both MMC- and BPDE-induced DNA lesions are bypassed by Pol κ in an error-free manner, which plays an important protective role against cell death induced by these chemicals (see the discussion below and Fig. 5).

Mutation spectrum analysis of the *TK* ^−/−^ mutants revealed that the expression of wild-type Pol κ suppresses mutations at CpG sites but not at GpG sites or other sequences (Fig. 3b), suggesting that Pol κ protects cells against MMC-induced genotoxic stresses via error-free TLS across the CpG inter-strand crosslinks at guanine bases in DNA. Two major pathways of DNA inter-strand crosslink repair at different cell cycle stages have been reported, replication-dependent or replication-independent repair [41]. In the replication-dependent repair process, the Fanconi anemia pathway is activated, resulting in the ubiquitylation of FANCD2-I [42], followed by unhooking of one side of crosslinking DNA strands by several nucleases, such as ERCC1-XPF and MUS81 [43]. On the other hand, in the replication-independent repair process, global genomic as well as transcription-coupled nucleotide excision repair (NER) pathway is known to be involved in resection of the duplex around crosslinking site [44, 45]. In both pathways, the unhooked crosslinked strand is bypassed by TLS Pols. Pol κ was supposed to be involved in the TLS process, but the fidelity of Pol κ-dependent DNA synthesis beyond the lesion had yet to be clarified in human cells [19–21, 46]. The results of the present study clearly demonstrate that Pol κ plays a substantial role in MMC-induced inter-strand crosslink repair through accurate TLS in human cells. Other TLS Pols such as Pol ζ and REV1 may also be involved in the TLS process but in an error-prone manner [46]. The involvement of Pol κ in the TLS suppresses both mutations as well as strand breaks in DNA because the LOH mutant in the *TK* gene mutation assay and the SCE incidence were significantly increased in KO TK+/- cells treated with MMC (Table 2 and Fig. 4b). In case that the MMC-induced DNA inter-strand crosslinks are not bypassed by TLS polymerases, the DNA lesions might cause replication fork collapse and double strand breaks, which can lead chromosomal alterations or cell death. Taken together, we suggest that Pol κ protects cells against MMC-induced genotoxicity through error-free TLS of CpG inter-strand crosslinks, resulting in the avoidance of mutation induction by other TLS Pols or cell death (Fig. 5).

Recently, it has been reported that *gpt* delta mice expressing catalytically inactive Pol κ exhibited an elevated frequency of mutations at CpG and also GpG sequences in bone marrow after MMC treatment [32]. The results presented here with CpG inter-strand crosslinks are consistent with the in vivo results and are also supported by biochemical evidence that Pol κ accurately bypasses the *N* ^2^-*N* ^2^ inter-strand crosslinks at CpG sites [19]. In contrast, there is a discrepancy with respect to mutation induction at GpG intra-strand crosslinks between the results in mice and human cells. The reason for this discrepancy is unclear, but one possible explanation is the difference in NER activity between Nalm-6-MSH+ cells and mice. Indeed, the *N* ^2^-*N* ^2^ intra-strand crosslinks induced by MMC, as well as cisplatin, could be substrates for NER [47]. Pol κ is supposed to function in a late step of NER as a gap-filling polymerase with replicate Pols [48], but this role appears to be more substantial under conditions of low nucleotide concentrations [49]. Nalm-6-MSH+ cells comprise a cancer-derived cell line generally believed to exhibit proficient nucleotide pools for continuous proliferation compared to normal cells. Therefore, we speculate that the MMC-induced GpG intra-strand crosslinks cannot be substrates for NER by Pol κ in Nalm-6-MSH+ cell lines; thus the mutation induction at the GpG site was similar between the WT TK+/- and KO TK+/- cells in this study. In fact, KO cells did not exhibit hypersensitivity to UV-C light in our previous study [26] despite the discovery of hypersensitivity in mouse ES cells [8, 49].

## Conclusions

In this study, we set up the cell lines for the *TK* gene mutation assay and also experimental condition for CA and SCE assays in a Nalm-6-MSH+ cell lines with different expression profiles of Pol κ. The cell lines can be used to evaluate the genotoxicity of chemicals from different scopes including gene mutation, chromosome aberration, and homologous recombination, in the same background. Using this cell lines, we found that Pol κ protects human cells against MMC-mediated genotoxicity by performing error-free TLS across CpG inter-strand crosslinks. This cell line could lead to a better understanding of the roles of Pol κ in terms of susceptibility to chemical carcinogenesis.

## Additional file

Additional file 1: Table S1. Primer list. **Table S2.** Mutation spectra of *TK* ^−/−^ mutants. (XLSX 23 kb)

## Abbreviations

BPDE: Benzo[*a*]pyrene-7,8-dihydrodiol-9,10-epoxide
CA: Chromosome aberration
CD: Nalm-6-MSH+ cell expressing catalytically dead Pol κ
KO: Pol κ-deficient Nalm-6-MSH+ cell
LOH: Loss of heterozygosity
MMC: Mitomycin C
NER: Nucleotide excision repair
PE: Plating efficiency
Pol: DNA polymerase
SCE: Sister chromatid exchange
TFT: Trifluorothymidine
TK: Thymidine kinase
TLS: Translesion DNA synthesis
WT: Wild-type Nalm-6-MSH+ cell
